# Oral Health Assessment in Prisoners: A Cross-Sectional Observational and Epidemiological Study

**DOI:** 10.3390/epidemiologia6040088

**Published:** 2025-12-05

**Authors:** William Alves dos Reis, Bruno Gomes dos Santos Martins, Rodrigo Resende, Urubatan Vieira de Medeiros, Juliana Campos Hasse Fernandes, Gustavo Vicentis Oliveira Fernandes

**Affiliations:** 1 Private Practice, Marietta, GA 30062, USA; 2 Surgery and Odontostomatology, University of Salamanca, 37007 Salamanca, Spain; 3 Department of Oral Surgery, Federal Fluminense University, Niteroi 24020-140, Brazil; 4 Department of Preventive Dentistry, Federal University of Rio de Janeiro, Rio de Janeiro 21941-617, Brazil; 5 Private Researcher, St. Louis, MO 63104, USA; 6 Missouri School of Dentistry & Oral Health, A. T. Still University, St. Louis, MO 63104, USA

**Keywords:** epidemiology, caries, periodontal disease, oral hygiene

## Abstract

Objectives: This cross-sectional observational and epidemiological study aimed to collect data on the oral health conditions of the prison population in Rio de Janeiro, Brazil. Methods: The Penitentiary Moniz Sodré, part of the Penitentiary Complex of Bangu, houses 1385 male inmates of different nationalities. They were divided into groups according to age: Group 1, prisoners aged 18 to 27 years; Group 2, from 28 to 37 years; Group 3, from 38 to 47 years; and Group 4, from 48 and older. A survey was performed, and the Decayed, Missing, Filled Teeth (DMFT) index was applied. A statistical analysis was conducted, considering a *p*-value of less than 0.05 as significant. Then, multiple linear regression was implemented to verify correlations among the studied parameters, to adjust for confounders, and to examine predictors of DMFT scores. Results: The average age was 26.95 ± 6.72 years, with 57.7% smokers (*n* = 720) and 7.7% (*n* = 96) former smokers. Lung diseases were also relatively common (20.9%). The most frequently reported oral issue was bleeding on probing (37.7%, *n* = 470), with 100% presenting visible dental plaque and 71.3% dental calculus. Oral hygiene habits showed moderate adherence, with 20.1% brushing their teeth at least once daily and 20.3% flossing; however, only 10.3% reported using fluoride mouthwash. The average DMFT score progressively increased across age groups: Group 1 (age: 18–27): 6.89; Group 2 (age: 28–37): 10.87; Group 3 (age: 38–47): 16; and Group 4 (age ≥ 48): 22.5 (*p* < 0.0001). Decayed (D) teeth scores showed a moderate increase: Group 1: 2.94 ± 2.74; Group 2: 3.38 ± 2.65; Group 3: 3.11 ± 2.56; Group 4: 3.75 ± 3.1 (*p* = 0.0029). Missing (M) teeth scores demonstrated a significant increase with age, from 2.74 (±2.84) in group 1 to 18.12 (±7.71) in group 4 (*p* < 0.0001), whereas Filled (F) teeth scores were highest in group 3 (mean 1.92 ± 4.13), followed by a decline in the oldest group (mean 0.62 ± 1.18) (*p* < 0.0001). These findings indicate a strong age-related increase in the total DMFT score, primarily driven by the number of missing teeth. Conclusions: High levels of caries and its sequelae exist, demonstrating a correlation with age, as well as a low level of previous periodontal treatment or intervention. As the treatments performed did not manage to reduce the incidence of caries and periodontal diseases, a high number of extractions were observed in patients in confinement.

## 1. Introduction

Health emphasizes the well-being of patients or populations. Therefore, historically, health services and professionals have focused on eradicating disease [1,2]. Health promotion is effective when implemented through continuous, serious, and supportive actions addressing factors such as diet, oral hygiene, and addictive habits, among all health professionals [3]. These aim to reverse the current situation and improve health conditions and quality of life (QoL) [4,5]. In dentistry, education on prevention and restorative care aim to provide the highest level of oral health, enabling a better quality of life [6,7]. Considering caries and periodontal disease, tooth brushing has been one of the most effective measures of prevention [8], which should be established as early as possible. However, patients are responsible for performing oral hygiene at home, as instructed by professionals [9]. Thus, dentists must patiently explain and convey the information and techniques using simple language to help and motivate patients to understand the disease, its origin and progression, and prevention methods [10,11]. The most crucial aspect of motivation is not the method but the professionals’ commitment and belief in what they are teaching [10,11,12].

Therefore, addressing these concerns in an incarcerated population is challenging worldwide. Research conducted in various penitentiaries shows that lack of resources and education regarding oral health significantly contributes to poor hygiene levels among these populations [13,14,15]. Specifically, the prevalence of dental caries and periodontal diseases is a significant concern within this population; studies have shown significantly poorer oral health compared to the general population [13,14,16]. For instance, a study conducted in a French prison reported that nearly 65% of inmates presented with periodontitis, indicative of the dire oral health situation within correctional facilities [17]. Consistent with these findings, studies in Indian prisons report high rates of dental caries, with prevalence rates soaring to 97.5% in some instances, alongside significant issues related to periodontal conditions [18,19].

Prisoners are particularly vulnerable to dental diseases due to limited access to oral healthcare, inadequate dietary provisions, and environment-related stressors. Evidence suggests that psychosocial factors directly and negatively influence oral health disparities and outcomes [19,20], highlighting the urgent need for comprehensive oral health education and accessible treatment options within correctional health systems [21,22,23]. In addition, prisoners’ oral health-related QoL is markedly compromised due to the oral diseases they face, which in extreme cases necessitates urgent dental interventions that prison facilities may struggle to provide [24,25]. Furthermore, a cross-sectional study indicated that drug addiction correlates with severe periodontal destruction among various populations [16].

Consumption of tobacco and high-sugar diets is common in prison settings, which aggravates dental problems. Heavy use of chewable tobacco among incarcerated women was found to be significantly associated with higher rates of dental caries and periodontitis [18]. Additionally, unhealthy dietary habits characterized by high caloric intake of sweets and refined carbohydrates have been linked to increased incidences of tooth decay and periodontal diseases among prisoners [18,20]. This pattern aligns with findings from other studies that illustrate the prevalence of periodontal disease in prisoners starkly contrasts with that in the general population, attributed to lifestyle factors [21,26].

The data/information available thus far presents a compelling picture of the oral health crisis within incarcerated populations, underlining substantial emerging trends that call for targeted public health interventions and policy reform to improve health outcomes for these at-risk groups. To address this concern, a rational program would help maintain and prevent oral diseases [4,27]; it would be focused on prevention while also addressing the diagnosis and treatment of existing lesions [28,29].

We acknowledge that data on local prisoners and convicts is rare in the literature. Thus, this cross-sectional observational study aimed to collect data on the oral health condition of the prison population, specifically dental caries and periodontal conditions, correlating them with age. The data gathered could facilitate the implementation of dental care services focused on health promotion and prevention, improving oral health not only during their confinement but also for the rest of their lives, thereby enhancing their physical, mental, and social well-being.

## 2. Materials and Methods

The study adhered to the Declaration of Helsinki (1964, updated 2024) and was previously approved by the local Institutional Review Board (IRB). All prisoners from the Moniz Sodré Penitentiary in Rio de Janeiro, Brazil, were invited to participate in the study. Those who accepted signed an informed consent form after receiving instructions on the participation process. All of them were in closed custody, with sentences ranging from 7 to 12 years in prison.

### 2.1. Characteristics of the Study

The present study is an observational epidemiological study in which the researchers are interested in the health–disease relationship occurring in the population and in appropriately expressing the respective frequencies. This data will reveal the needs and characteristics of the segments that could benefit from preventive and prophylactic measures, making it easier to provide the necessary oral care for prisoners.

### 2.2. Samples and Groups

Within the Bangu Penitentiary complex, there is a specific penitentiary, the Moniz Sodré penitentiary, which serves as a model for the Bangu Penitentiary complex. Those receiving dental assistance are permitted to reduce disruptions to their daily routines during treatments, facilitating data collection. The Moniz Sodré Penitentiary was chosen through a simple sampling system. The groups were categorized by age into the following groups: Group 1, prisoners aged 18 to 27 years; Group 2, prisoners aged 28 to 37 years; Group 3, prisoners aged 38 to 47 years; and Group 4, prisoners aged 48 and older.

### 2.3. Data Collection

Data collection was conducted by one author (W.A.R.) and trainees from the SEJDH Health Superintendence, who were supervised by him and had previously been calibrated for the study (*k* = 0.896). Anamnesis forms, which included medical and dental records (such as educational level, general health, and oral condition), were completed. The Decayed, Missing, Filled Teeth (DMFT) index [30] indicates the number of permanent teeth affected by caries. In the individual examined, it is the total of the number of “filled” teeth, the number of teeth with cavities, and the number of missing teeth. Therefore, in the permanent dentition, its values range from 0 “zero” (no teeth infected with caries) to 32 (all teeth infected with caries); as caries tends to increase with age, the DMFT was calculated at each age. General data on prisoners’ oral health (anamnesis) was collected to acquire knowledge of current complaints, habits, past dental treatments, oral health, and general health behaviors.

### 2.4. Statistical Analysis

A descriptive statistical analysis was conducted to evaluate the distribution of categorical variables. Data were tabulated and analyzed using SPSS software (v. 11.0, SPSS Inc., Chicago, IL, USA). The Shapiro–Wilk test was applied to assess the normality of continuous variables. For comparisons of means across groups, one-way ANOVA (Analysis of Variance) was employed, with statistical significance set at *p* < 0.05. Subsequently, multiple linear regression analyses were performed to explore associations among the studied parameters and to identify potential predictors of DMFT scores. These models were adjusted for relevant potential confounders, such as smoking status and education level, to account for their possible influence on the outcomes. Where applicable, univariable regressions were also conducted for comparison.

## 3. Results

**Figure 1:**
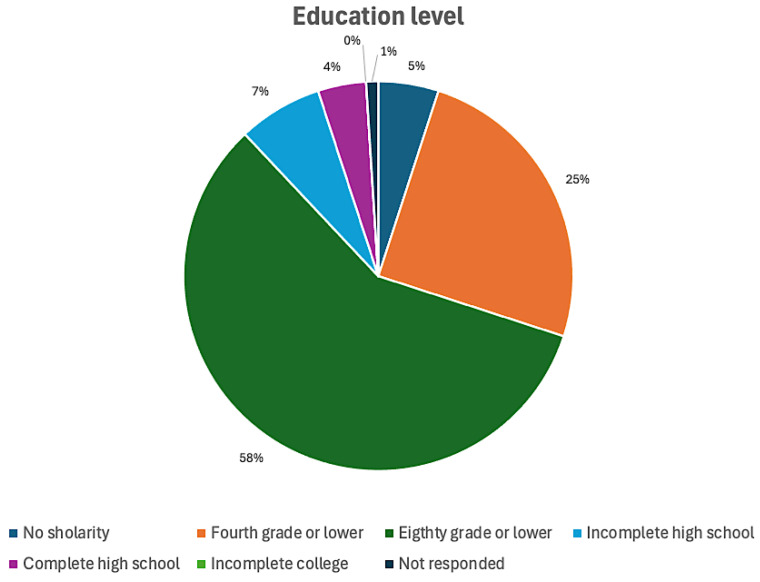
The graphic shows that the majority of those examined, 58% (*n* = 803), had studied up to the 8th grade, while 25% (*n* = 346) had studied up to the 4th grade of elementary school. Additionally, none of the examined individuals had started college.

The number of inmates in the penitentiary at the time was 1385, all men from various ethnic groups, with a predominance of Afro-descendants and multiple nationalities. They undergo regular medical and dental examinations and receive a set of toothbrushes and toothpaste from the prison every three months. Intervention procedures are limited to basic care and support. Figure 1 shows the educational level of the participants.

**Figure 2:**
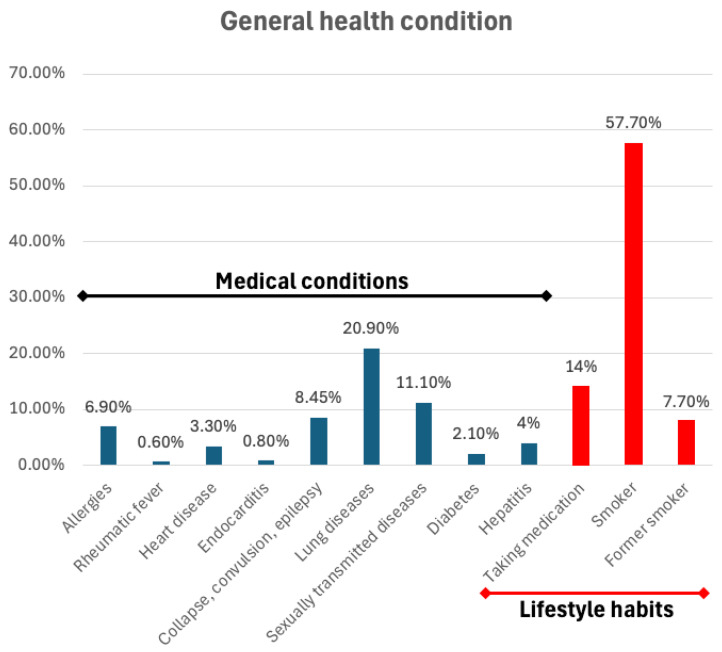
General health condition (medical conditions in blue/black; lifestyle habits in red). The graphic shows that 57.70% were smokers and 7.7% were ex-smokers, with 20.9% presenting with lung diseases, such as tuberculosis; 11.1% have or have had sexually transmitted diseases.

The data shown in Figure 2 summarizes the overall health status of the studied population. In terms of general health, a significant proportion of individuals reported being smokers (57.7%, *n* = 720), with a smaller percentage identifying as former smokers (7.7%, *n* = 96); interestingly, lung diseases were also relatively common (20.9%). The use of any daily medication (14%) and a history of sexually transmitted diseases (11.1%) were reported for a considerable number of inmates. Other conditions, such as collapse, convulsions or epilepsy (8.45%), and heart disease (3.3%), were less frequently reported, while diseases like rheumatic fever, endocarditis, and diabetes showed notably lower prevalence rates, each under 3%.

**Figure 3:**
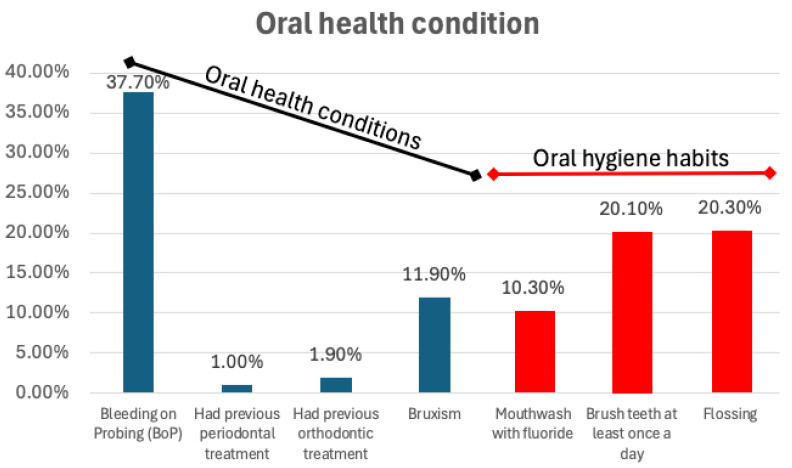
Oral health condition (oral health conditions in blue/black; oral hygiene habits in red). The graphic shows a high level of bleeding on probing (BoP) (37.7%) detected; 20.3% of the inmates reported flossing, and 20.1% brush their teeth at least once daily.

Regarding oral health (Figure 3), the most common issue was bleeding on probing (BoP), affecting 37.7% of the population (*n* = 470), which indicates a high level of gingival inflammation or periodontal disease. Surprisingly, only 1% had previously undergone periodontal treatment, and just 1.9% had a history of orthodontic treatment, suggesting limited access to or utilization of dental care. Oral hygiene habits showed moderate adherence, with 20.1% brushing their teeth at least once daily and 20.3% flossing. However, only 10.3% reported using fluoride mouthwash, which may highlight a gap in preventive dental practices.

The oral hygiene routine in correctional facilities is often limited, with most inmates brushing their teeth only once or twice a day, typically in the morning and evening, without supervision or standardized oral hygiene education. Access to toothbrushes, toothpaste, and dental floss may be restricted or inconsistent. Furthermore, the institutional diet tends to be carbohydrate-rich and low in fresh fruits and vegetables, often containing processed or sugary items. In addition, the most consumed liquids are water and sweetened beverages such as processed juice or sugary coffee.

Additionally, bruxism was reported by 11.9% of the participants, which could be linked to stress or occlusal issues. The overall findings indicate a population with significant health risks, especially related to smoking and respiratory conditions, as well as suboptimal oral hygiene practices and a low prevalence of dental care utilization. These insights underscore the importance of targeted health education and enhanced access to both medical and dental services. The clinical assessment revealed that 100% of the examined individuals had visible dental plaque, and 71.3% had dental calculus.

Table 1 summarizes the DMFT index (according to WHO criteria) by age group. The average DMFT score steadily increased across age groups, with values of 6.89 (±4.82) in the youngest group and 22.5 (±6.71) in the oldest group. Decayed (D) teeth scores showed a moderate increase from 2.94 (±2.74) in group 1 to 3.75 (±3.1) in group 4 (*p* = 0.0029). Missing (M) teeth scores increased significantly with age, from 2.84 (±2.27) in group 1 to 18.12 (±7.71) in group 4 (*p* < 0.0001). Filled (F) teeth scores were highest in group 3 (mean 1.92 ± 4.13) and then decreased in the oldest group (mean 0.62 ± 1.18) (*p* < 0.0001).

The high proportion of decayed teeth (D) observed in the DMFT scores among incarcerated individuals, particularly with progression across age groups, highlights significant deficiencies in access to timely and effective dental care within the prison system. In most correctional facilities, dental services are limited in both availability and scope. Routine preventive care, such as regular dental check-ups, prophylaxis, fluoride application, and restorative procedures, is often not systematically provided. Dental care is typically reactive rather than preventive, with services largely restricted to emergency-based interventions focused on pain relief or infection control. As a result, tooth extractions are frequently the primary treatment modality, especially when restorative materials or time are limited. This contributes to a cycle of untreated caries and tooth loss over time, particularly among older inmates who may have accumulated unmet treatment needs during prolonged incarceration. Furthermore, the limited availability of dental professionals and the absence of individualized oral health education contribute to the persistence and progression of untreated decay. The institutional environment, as previously noted, includes inadequate oral hygiene routines, restricted access to dental hygiene products, and a cariogenic diet, all of which exacerbate oral health deterioration. Taken together, these factors help explain the high and progressive “D” component in the DMFT index among prisoners and underscore the need for systemic improvements in prison dental care, including preventive strategies, timely restorative treatments, and health education tailored to this vulnerable population.

The statistical analysis examined the relationship between age and dental health outcomes, specifically the number of decayed (D), missed (M), and filled (F) teeth, as well as the overall DMFT (Decayed + Missed + Filled Teeth) score. Regarding the correlation between D and age, a significant positive association was observed between age and the number of decayed teeth, with a regression coefficient of 0.0065 (*p* = 0.012). It indicates that for each additional year of age, the number of decayed teeth increases slightly by a small amount. While this effect size is modest, it is statistically meaningful, with *p* ranging from 0.001 to 0.012.

For M and age, a strong positive association was observed. The coefficient was 0.3964 (*p* < 0.001), indicating that each additional year of age is associated with a 0.40 increase in the number of teeth lost. The high *t*-value (75.71) and narrow confidence interval (0.386 to 0.407) demonstrate the strength and precision of this association.

Otherwise, the correlation between F teeth and age was negatively associated. The coefficient was −0.0111 (*p* < 0.001), indicating that as people age, they tend to have fewer filled teeth. This could indicate less restorative dental care or more advanced disease progression with age. The *p*-value ranged from −0.016 to −0.006, confirming this inverse relationship.

The total DMF (D+M+F) showed a robust positive correlation with age. The coefficient was 0.4910 (*p* < 0.001), indicating that each year of age corresponds to nearly half a unit increase in the combined total of D, M, and FT. The very high t-value (78.38) and narrow confidence interval (0.480 to 0.502) highlight the strength of this relationship.

These findings collectively highlight the growing burden of dental disease as people age, emphasizing the importance of preventive care throughout the lifespan. Below is a summary of the regression results regarding the association between age and dental health outcomes (Decayed, Missed, Filled, and total DMF) (Table 2).

## 4. Discussion

The goal of this cross-sectional epidemiological study was to collect oral health data from the prison population to assess dental care services, with a focus on health promotion and prevention. This analysis aims to identify ways to improve oral health during confinement and in public and social life, thereby enhancing physical, mental, and social well-being.

### 4.1. Limitations of Oral Health Epidemiology in Adults and Inmates

Epidemiological data on oral health are usually limited to schoolchildren, as they are typically grouped for research purposes. Data on the adult population is restricted due to social life, which makes it challenging to collect a representative sample of the population in suitable locations for conducting studies. In addition, the epidemiology of dental caries and periodontal conditions in incarcerated populations has become an important area of research due to the unique health challenges faced by prisoners.

### 4.2. Oral Health Studies in Specific Populations

Several studies have been published recently assessing the oral health of specific populations, such as children with oral clefts [31], indigenous people [32], populations with mental disorders [33,34], adolescents [35], and children with autism spectrum disorder [36,37]. Although data on the evolution of DMFT exist, few studies have been conducted in adult populations in Brazil, making direct comparisons of data difficult. Some studies have been published in the past decade, but only a few contain directly available DMFT data. Education, socioeconomic status, and oral hygiene habits have been associated with these findings [38].

Data reveal that even in higher-educated populations and families with higher socioeconomic status, the findings of this study regarding oral hygiene habits and literacy remain a concern in the opposite direction [39,40]. Social disparities and social risk factors are key issues across all studies, affecting the DMFT and oral health status [41].

### 4.3. Sociodemographic and Health Characteristics and Findings, and DMFT Trends

Regarding the present study, the statistics on education revealed that most of those examined (approximately 58%) had completed up to the 8th grade, 25% had completed up to the 4th grade, and none of those had begun college.

The results regarding general health are noteworthy, indicating that 57.7% of those examined are smokers and an additional 7.7% are ex-smokers. Furthermore, 20.9% of the participants have lung diseases (e.g., tuberculosis), and 11.1% have or have had sexually transmitted diseases.

Regarding the oral health results, it was found that 37.7% of those examined had significant BoP, with bacterial plaque present in all participants. Additionally, 20.3% reported using dental floss, and 20.1% brushed their teeth at least once a day. It also indicated a high prevalence of dental caries among those examined, particularly among individuals with a low level of previous or a history of periodontal treatment.

The dental health status of participants in different age intervals showed that the older the participant, the higher the risk of having decayed, missed, or filled teeth. The increasing trend in the DMFT index with age reflects the cumulative effect of decay and tooth loss over time. Moreover, the high standard deviations observed in the older age groups (e.g., 7.71 for missed teeth in the ≥48 group) suggest a high level of variability in dental health among individuals in these categories. This variation highlights the impact of factors such as the willingness to seek dental care, oral hygiene habits, and educational status, which can influence dental health outcomes.

Data concerning DMFT has evolved in recent years. In Brazilian adults aged 35 to 44 in 1986, the DMFT was 22.5, decreasing to 20.1 by 2000 and further to 16.3 by 2010. These numbers align with the findings of this study. In 2023, some authors [42] reported a drastic drop in DMFT assessment, which was 7 ± 4.5; this number is considerably lower than those found in the present study and is in line with the country-wide trend of DMFT reduction. The populations in both studies are vastly different, as the prison population (in the present study) primarily had lower education levels and faced a distinctly different social scenario.

### 4.4. Influence of Institutional Diet on Oral and Systemic Health

The contemporary institutional diet, particularly in prison settings, is often characterized by a high intake of carbohydrates while lacking sufficient fresh fruits and vegetables. Such diets frequently contain processed foods and sugary items, which can contribute to various health issues. High-carbohydrate diets have been associated with elevated plasma triglyceride levels and insulin resistance, which are particularly concerning for individuals at risk of conditions like Type 2 Diabetes Mellitus [43,44].

For instance, carbohydrate-rich diets can significantly influence lipid metabolism, resulting in increased triglyceride levels that may heighten the risk of hyperlipidemia and insulin resistance [43]. Additionally, sweetened beverages, especially juices and sodas, are commonly consumed in these settings. These drinks are high in sugar and lacking in nutritional value, leading to excessive caloric intake without beneficial nutrients [45]. The consumption of such sweetened beverages is linked to weight gain, negative metabolic consequences, including insulin insensitivity, and high levels of decay, which contributes to unhealthy dietary cycles that can result in long-term health complications [46,47].

### 4.5. Impact of Alcohol and Drug Use on Oral Health

Alcohol and drug consumption have detrimental impacts on dental health. Moldvai et al. [48] identified a correlation between poorer oral health, reflected in higher DMFT values, and factors such as older age, diabetes, tobacco smoking, and low socioeconomic status among stroke patients, which also implicates alcohol and drugs as contributing risk factors.

Additionally, Sheth et al. [49] noted that the consumption of alcohol and tobacco may modulate salivary cytokines, leading to an increase in dental caries and a higher DMFT index among users due to diminished oral health quality. Giudice et al. [50] found that patients undergoing methadone treatment, associated with drug use, demonstrated significantly worse oral health, indicated by elevated DMFT scores arising from neglected dental hygiene linked to drug dependence. Collectively, these studies highlight the complex interplay between substance use and oral health, illustrating how drugs and alcohol can exacerbate dental diseases and contribute to a higher prevalence of DMFT.

### 4.6. Comparative Studies on Prisoners’ Oral Health Worldwide

Studies consistently show that inmates exhibit a higher prevalence of dental caries compared to the general population. For instance, a study conducted in Chennai (India) reported a notably high prevalence of dental caries (58.2% of males and 54.2% of females), especially among younger prisoners, and a mean DMFT index of 5.1 and 3.9 for female and male prisoners, respectively, emphasizing the urgent need for targeted oral health interventions within this demographic [51]; those numbers were lower compared to the present study.

A similar investigation in South Africa found that 66.4% of individuals using public health services presented with dental caries, the most prevalent oral condition, followed by periodontal disease (11.7%), highlighting the pressing oral health crisis in under-resourced populations, including prisoners [52].

These findings are echoed by research on juvenile prisoners (mean age of 16.33 years) [53], where limited access to dental care and various socioeconomic challenges contribute to a higher incidence of dental issues, including untreated decay and tooth loss. The DMFT scores found were 9.09 for 78 juvenile prisoners aged 16–17 years, followed by 9.0 for 19 prisoners aged 14–15 years and 7.13 for eight prisoners aged 12–13 years—higher numbers than those found in the present study. The oral hygiene status of the prisoners revealed that more than half of the juvenile prisoners (53.3%) had poor oral hygiene, while less than half (45.7%) had fair oral hygiene; similar data were found in the present study.

Additionally, a systematic review and meta-analysis focusing on inmates in India indicated that the overall dental health was poor, with a mean DMFT score of 3.9 for male prisoners, while female prisoners exhibited even higher scores at 5.1, with the pooled prevalence of caries among prisoners being 78.42% (59.48–92.58%) [19]. This finding aligns with another study, which reported that approximately 79% of prisoners in a district jail displayed poor periodontal conditions, alongside significant incidences of periodontal diseases and dental fluorosis, and a pooled prevalence of depression among prisoners of 48.78% (95% CI: 27.24–70.55%) [54]. The authors observed that poor mental and dental health standards and the virtual absence of healthcare facilities necessitate governmental actions to boost inmates’ health.

In jurisdictions such as France, similar trends were observed; a retrospective study reported that 93% of inmates had at least one decayed untreated tooth, 95% had periodontal disease, and the use of psychoactive drugs seemed strongly related to oral health status degradation [17].

### 4.7. Parallels with Military Populations

When comparing dental health within the prison population to military personnel, there are noteworthy parallels. Military populations face unique oral health challenges primarily due to lifestyle, stress levels, and access to dental care. A longitudinal study involving military personnel reported similarly high rates of periodontal disease (28% of the teeth), with 58.8% presenting mild periodontal pockets and 37.1% severe periodontal pockets (averages of probing depth and clinical attachment loss [CAL] were 2.17 and 2.36 mm—52% with mild CAL and 47.5% with severe CAL; plaque and gingival bleeding indices were 71% and 40.3%; BoP of 86.5%) [55,56].

A systematic review corroborated the notion that both incarcerated individuals and military personnel encounter significant oral health disparities arising from their respective environments [57]. Understanding these parallels can help frame effective preventive measures and interventions across both closed populations, considering their shared challenges in accessing comprehensive oral healthcare.

Furthermore, a study from Finland found that a high percentage of inmates engaged in substance abuse, which correlated with elevated levels of oral health issues like periodontal disease [15]. Substance use disorders, common in both prisoners and military personnel, exacerbate dental problems, leading to higher rates of tooth loss and progressive periodontal disease [18]. Moreover, research in Brazil revealed that many prisoners suffered from untreated dental caries, mainly attributable to their limited educational and health literacy levels, which is a commonality observed in both incarcerated individuals and particular military cohorts [24].

### 4.8. The Role of Education and Professional-Patient Interaction

A professional needs to clarify the etiology of the disease or disorder in question and the methods that can be used to treat, control, and cure it, as well as the necessary preventive measures. Honest communication, a genuine interest in the patient’s well-being, and the use of psychological procedures and techniques that aim to foster the patient’s collaboration all favor the treatment process, as well as the development of a trusting relationship [58].

A fundamental step in establishing a positive interpersonal relationship between a dentist and patient is the development of the patient’s trust in the professional and themselves [59]. The educational process in dentistry is utilized to modify behaviors necessary for maintaining, acquiring, and promoting health. To help patients learn how to maintain good health, it is not enough to explain the causes of diseases and how to avoid them; they must also be encouraged to take an active role in their own learning.

It is necessary to create the will to learn, arouse attention, and create the essential interest that triggers action. It stimulates their desire to achieve the desired results, fosters internal conditions favorable to learning, and makes it a pleasure. As a result, individuals dedicate the best of their time and effort to it [5].

### 4.9. Limitations of the Study and Future Directions

The ideal evaluation of the data would be better debated in conjunction with other results found in the literature. A limitation of the study was its development in a single institution setting, which limited the sample size and introduced potential biases from the self-reported data collected. Therefore, the results indicate that the outcomes were high, suggesting that dentistry in the prison system has been fundamentally restorative, as evidenced by the findings. Although we acknowledge that this approach cannot fully control oral diseases, it is evident that the results were positive; what happens in the eminent curative and restorative practice perpetuates a repetitive cycle of treatments without managing to reduce the incidence of caries and periodontal diseases, leading to a high number of extractions in patients in confinement.

The proposal to change professionals’ attitudes, combined with planning for managing oral health services and focusing on health promotion practices in prisons, while always considering the laws of the Brazilian National Health Service (*Sistema Único de Saúde* [S.U.S.]), will ultimately bring about significant changes in the epidemiological landscape of oral diseases. New strategies could be explored for systemic and oral conditions, including biotics (such as prebiotics and probiotics) [60,61,62], other medications [63], and digital technologies [40].

## 5. Conclusions

Within the limitations of this study, this study reveals a 100% prevalence of dental caries among incarcerated individuals, with worsening severity as age increases. The high number of decayed (D) and missing (M) teeth components, alongside very few filled (F) teeth, indicates limited access to restorative care and a reliance on extractions as the primary treatment. Additionally, gingival inflammation (37.7% BoP)/periodontal diseases and poor oral hygiene habits (low brushing and flossing activities, and fluoride use) highlight systemic neglect of preventive care in prison dentistry.

Study limitations, including single-institution data and self-reported biases, underscore the need for more comprehensive research. However, the findings demonstrate that the current curative–restorative model is ineffective in controlling oral diseases, perpetuating a cycle of decay and tooth loss. To break this cycle, urgent reforms are needed and recommended: (1) preventive programs (fluoride varnish, supervised brushing, dietary counseling); (2) structured oral health education (digital literacy, hygiene workshops); (3) policy changes (expanding restorative care under Brazil’s S.U.S., integrating mobile dental units); and (4) long-term monitoring to assess intervention effectiveness. A shift from reactive to preventive dentistry, aligned with public health principles, could reduce extractions, improve inmate health, and lower long-term costs. Future efforts must also engage families and communities to reinforce behavioral changes beyond incarceration. Without systemic change, the epidemic of dental diseases in prisons will persist. Therefore, new research is recommended to expand the present study, allowing for a more comprehensive comparison of the variables and confounding factors identified. Additionally, it is suggested that an investigation be conducted into the relationship between variables and diseases with underlying conditions related to incarceration, also comparing the results of programs implemented, as previously recommended.

## Figures and Tables

**Table 1:** DMFT index (WHO) by group.

Group	Age Interval	n	Age	± SD	Decayed (D)	± SD	Missed (M)	± SD	Filled (F)	± SD	DMFT (Sum)	± SD
1	18–27	889	23.21	2.5	2.94	2.74	2.84	2.27	1.09	2.27	6.89	4.82
2	28–37	388	31.04	2.71	3.38	2.65	5.82	3.06	1.7	3.06	10.87	6.33
3	38–47	83	42.3	2.99	3.11	2.56	10.5	7.04	1.92	4.13	16	7.64
4	≥48	25	53.5	3.89	3.75	3.1	18.12	7.71	0.62	1.18	22.5	6.71
F-statistic			-		4.69		363.2		17.07		147.96	
*p*-value			-		0.0029		<0.0001		<0.0001		<0.0001	

DMF (Sum) = permanent teeth D+M+F; D = decayed; M = missed; and F = filled teeth.

**Table 2:** Linear regression analysis of dental health outcomes by age.

Outcome Variable	Coefficient (β)	Std. Error	*t*-Value	*p*-Value	95% Confidence Interval	Interpretation
Decayed (D)	0.0065	0.003	2.52	0.012	[0.001, 0.012]	Significant positive association: Older age is slightly associated with an increased number of decayed teeth.
Missed (M)	0.3964	0.005	75.71	<0.001	[0.386, 0.407]	Strong positive association: Each year of age increases the number of missed teeth by ~0.40.
Filled (F)	−0.0111	0.003	−4.21	<0.001	[−0.016, −0.006]	Negative association: Increasing age is linked to fewer filled teeth.
DMF (D+M+F)	0.4910	0.006	78.38	<0.001	[0.480, 0.502]	Very strong positive association: Each additional year contributes ~0.49 more total DMF.

Note: All models are adjusted for intercepts. DMF = Decayed, Missed, and Filled teeth.

## Data Availability

The original contributions presented in this study are included in the article. Further inquiries can be directed to the corresponding author.
